# Chronic corticosterone exposure disrupts hepatic and intestinal bile acid metabolism in chicken

**DOI:** 10.3389/fvets.2023.1147024

**Published:** 2023-05-17

**Authors:** Lei Wu, Xinyi Liu, Aijia Zhang, Huimin Chen, Ruqian Zhao, Yimin Jia

**Affiliations:** ^1^Key Laboratory of Animal Physiology and Biochemistry, College of Veterinary Medicine, Nanjing Agricultural University, Nanjing, China; ^2^Jiangsu Collaborative Innovation Center of Meat Production and Processing, Quality and Safety Control, Nanjing, China

**Keywords:** chronic stress, glucocorticoids, fatty liver, bile acid, gut microbiota

## Abstract

**Objective:**

Chronic stress leads to a high circulating level of glucocorticoids, which disrupts lipid metabolism and causes non-alcoholic fatty liver disease in mice and humans. Meanwhile, bile acid (BA), a class of metabolites initially synthesized in the liver and further metabolized by gut microbiota, plays a vital role in lipid metabolism. This study aimed to investigate the effects of glucocorticoids on BA metabolism and gut microbiota in chickens.

**Methods:**

In this study, 35-day-old chickens were injected with 4 mg/kg/day corticosterone (Cort) for 14 days to simulate chronic stress.

**Results:**

Cort treatment significantly increased the triglyceride contents in the plasma and the liver. HE and oil-red staining showed that Cort treatment induced fatty liver in chickens. Meanwhile, Cort exposure downregulated total bile acid (TBA) content in the liver while increasing the TBA in feces. UPLC-HRMS results showed that Cort exposure significantly decreased the hepatic levels of CDCA, T-alpha-MCA, and T-beta-MCA. Moreover, Cort exposure significantly reduced the expression of genes related to BA synthesis (CYP8B1 and CYP27A1), conjugation (BACS), and regulation (KLβ and FGFR4). 16s sequencing results showed that Cort treatment significantly decreased the amount of *Lachnospiraceae, Eisenbergiella, Blautia*, and *Eubacterium* and increased the abundance of *Barnesiella, Lactobacillus*, and *Helicobacter*. Spearman correlation analysis showed a significant positive correlation between fecal TBA and the abundance of *Lactobacillales, Lactobacillus*, and *Barnesiella*. In comparison, TBA in the liver was positively correlated with *Eubacterium*, and negatively correlated with *Helicobacter*.

**Conclusion:**

In summary, chronic Cort exposure disrupts hepatic and intestinal bile acid metabolism inducing gut microbiome dysbiosis, which might associate with the development of fatty liver in chickens.

## Introduction

Nowadays, poultry is a flourishing industry all over the world including in China. To improve economic efficiency, intensive farming is increasingly popular in the poultry industry. This farming model exposes the chickens to constant stressors, such as high humidity/temperature, noise, and continuous illumination, which seriously decreased weight loss, increased feed conversion rate, and induced peripheral fat deposition. Chronic stress can increase the secretion of glucocorticoid (GC), a stress hormone produced by the adrenal cortex, and plays regulatory roles in physiological processes, such as growth, development, reproduction, and immunity (1). Previous studies have shown that chronic corticosterone (Cort) treatment can promote the hepatic deposition of triglycerides (TG) in chickens, leading to severe liver steatosis (2, 3). Moreover, clinical studies show that excessive GC-induced liver steatosis accounts for large numbers of patients with fatty liver (4, 5). However, the detailed underlying mechanism of how GC regulates lipid metabolism is still unclear due to the complexity of lipid metabolism.

Bile acid (BA), the dominant final product of cholesterol metabolism, is synthesized in the liver and excreted into the small intestine for further catabolism by the gut microbiota (6, 7). BA homeostasis is tightly regulated by a network of genes, including BA synthesis-associated genes [e.g., cholesterol 7-α hydroxylase (CYP7A1), sterol 27-α hydroxylase (CYP27A1), oxysterol 7-α hydroxylase (CYP7B1), and microsomal sterol 12-α hydroxylase (CYP8B1)], BA conjugation-related genes [bile acid: CoA synthetase (BACS) and bile acid-coenzyme A: amino acid N-acyltransferase (BAAT)], BA transportation-related genes [organic solute transporter α/β (OSTα/β), multidrug resistance-associated protein 2 (MRP2), and bile salt export pump (BSEP)], and enterohepatic circulation-related genes [organic anion co-transporting polypeptides (OATP), Na+/taurocholate co-transporting polypeptide (NTCP), ileal bile acid-binding protein (IBABP), apical sodium-dependent bile salt transporter (ASBT)] (8). Generally, BA functions as an emulsifier for intestinal lipid and fat-soluble vitamin absorption. In addition, BA is also a signal molecule binding to nuclear receptors in the liver and intestine, farnesoid X receptor (FXR), and Takeda G protein-coupled receptor 5 (TGR5) to regulate lipid metabolism (8, 9). Emerging studies show that disrupted BA homeostasis affects hepatic lipid and energy metabolism, contributing to the pathogenesis of metabolic diseases [e.g., diabetes, obesity, and non-alcoholic fatty liver disease (NAFLD)] (9–12).

On the other hand, the gut microbiota could metabolize BA *via* numerous reactions, including hydroxylation by bile salt hydrolases (BSH), epimerization, oxidation, and esterification (6). It has shown that depletion of gut microbiota alters BA homeostasis, such as elevated intestinal BA and decreased diversity of hepatic BA. While BA also functions as an anti-microbial agent, disrupting the bacterial membrane to alter its macromolecular structures through detergent actions (13). Thus, the BA levels could also affect the gut microbiota composition (14). For instance, taurine-conjugated BA promotes the proliferation of some specific bacteria strains (15). Since fatty liver disease is related to alterations of BA homeostasis and gut microbiota dysbiosis, the interaction between BA metabolism and gut microbiota and its impact on the pathogenesis of fatty liver is of great interest. Although the interplay among BA metabolism, gut microbiota, and fatty liver is evident, the directionality and causality of the interaction are still unclear.

It has shown that long-term treatment of GC caused lipid accumulation and changed gut microbiota structure in rats (16). Our previous study shows that chronic Cort treatment leads to fatty liver in chickens (2). However, whether the BA metabolism and gut microbiota are affected by Cort treatment in chickens remains to be determined. Thus, this study aims to investigate chronic Cort exposure's effect on BA metabolism and gut microbiota and clarify the correlation and possible underlying mechanisms. The findings of this study are anticipated to provide insights into improving broilers' welfare by alleviating chronic stress's impact on production performance in the intensive farming system.

## Materials and methods

### Animals and treatment

Animal protocols were approved by the Animal Ethics Committee of Nanjing Agricultural University, with project number 2012CB124703. The sampling procedures comply with the “Guidelines on Ethical Treatment of Experimental Animals” (2006) No. 398 set by the Ministry of Science and Technology, China.

One-day-old male broiler chickens (Xueshan Grass chickens) were obtained from a commercial hatchery (Lihua, Jiangsu, China). All chickens were individually weighed, wing labeled, and raised according to the procedure. Environmental conditions such as humidity, temperature, illumination, and ventilation were controlled during the experiment, depending on the age of the chickens (35–37°C during 1st week, and decreased 3°C per week until reaching 21°C at 5 weeks old). Twenty-four chickens (5-week-old) were randomly selected and divided into the solvent (Con) and corticosterone (Cort) groups, which were daily subcutaneously injected with either solvent (15% ethanol) or corticosterone (4.0 mg/kg body weight twice a day, 8:00–9:00 am and 17:00–18:00 pm) for 14 days.

At 49 days, all the chickens were weighed and killed by rapid decapitation, which complies with the American Veterinary Medical Association (AVMA) Guidelines for the Euthanasia of Animals: 2013 Edition. Blood samples were collected into EDTA-coated tubes and centrifugal to separate plasma samples. Liver samples were cut into small cubes and fixed in 4% paraformaldehyde for further histological analysis. Liver, cecal contents, and ileal mucosa were collected and frozen in liquid nitrogen. At the end of the necropsy, all the samples were saved under −80°C for further analysis.

### Hematoxylin-eosin (HE) and Oil Red staining

To evaluate the histological characterization of fatty liver in chicken, we performed HE and Oil Red staining for lipid deposition in hepatocytes by SeverceBio Inc. (Wuhan, China). Briefly, place the slides with liver tissue sections into filtered Harris Hematoxylin solution for 10 s. After washing with deionized water, immerse sections into EOSIN staining solution for 30 s. Wash the slides with deionized water and followed various steps of dehydration in ascending alcohol solutions (50, 70, 80, 95% × 2, 100% × 2). Clear the slides with xylene and mount the coverslip onto the section with Permount (Fisher, USA). All the histological slides were examined and images were taken by light microscopy (OLYMPUS, Japan).

### Determination of plasma glucose, total cholesterol, TG, HDL, and LDL

The plasma glucose, total cholesterol (Chol), triglyceride, high-density lipoprotein (HDL), and low-density lipoprotein (LDL) were determined by an automatic biochemical analyzer (HITACHI-7020) using commercial kits (H108, H201, H202, H203, and H207) according to the manufacturer's instructions.

### Measurement of hepatic Chol and TG

Hepatic triglyceride (TG) and Chol were determined by using a commercial assay kit (E1013 and E1015, Applygen Technologies Inc., China) following the manufacturer's instructions.

### Determination of total bile acids in plasma, liver, and cecal digesta

Total bile acids (TBA) in the liver and cecal digesta were extracted as previously described (17). Approximately 100 mg liver and 200 mg dry cecal digesta were homogenized in 1 mL 95% ethyl alcohol (EtOH) and incubated under 60°C overnight. After 12 h incubation, the homogenate was centrifuged at 8,000 rpm for 10 min to collect the supernatant. Then, the precipitate was resuspended in 1 mL 80% EtOH and incubated under 60°C overnight for the second extraction. The second supernatant was collected, and the precipitate was resuspended in 1 mL 2:1 (v:v) chloroform: methyl alcohol for 12 h under room temperature. The third supernatant was collected and mixed with the previous two tubes of supernatants for further TBA analysis. Plasma and previously extracted supernatants from the liver and cecal digesta were subjected to determine TBA by the automatic biochemical analyzer (HITACHI-7020, Hitachi, Japan) using a commercial kit (OR101) following the manufacturer's instructions.

### Bile acid analysis

The bile acid profiles in the liver were quantified as previously validated UPLC-Q/Orbitrap-HRMS procedures (18, 19). Briefly, 100 mg liver was homogenized in 0.5 mL deionized water and then centrifuged at 20,000 *g* for 10 min at 4°C. The supernatant was separated and evaporated with a vacuum centrifugal concentrator. The residue was resuspended in 100 uL methanol (80% v/v) and then filtered by a 0.22 μm nylon syringe filter. The activated carbon adsorption method prepared the blank and positive controls. The mobile phase flow rate was 0.2 ml/min, and the injection volume was 5 μL. The gradient elution was optimized to separate the different components of bile acids as previously described. A negative selective ion monitoring mode was selected to acquire all mass spectrometry data, which were analyzed by the Xcalibur 4.0 software. The above BA analysis was carried out by BioNovoGene, Inc. (Suzhou, China).

### RNA isolation and real-time PCR

Thirty microgram liver samples and 20 mg ileal mucosa samples were used to isolate total RNA using 1 mL TRIzol Reagent (Invitrogen, USA). Total RNA was reverse-transcribed to cDNA, which was subjected to real-time PCR with an Mx3000P Real-Time PCR System (Stratagene, USA). The primers (Supplementary Table 1) were synthesized by Tsingke Biotech (Nanjing, China). 18s RNA was used as an internal control. All samples were run in duplicates. Data were analyzed using the method of 2^−Δ*ΔCt*^.

### Western blotting

Frozen liver tissues were homogenized in a RIPA lysis buffer with 1% protease inhibitor cocktails (Bimake, Shanghai, China). After centrifugation at 12,000 *g* for 15 min, the supernatant was transferred into another tube. Then, the protein concentrations were determined by a commercial BCA protein quantitative kit (Biosharp, China), and 20 μg extracted protein was separated by SAS-PAGE and transferred to a nitrocellulose membrane for further western blotting analysis. The primary antibody CYP27A1 (14739-1-AP, dilution 1: 1000) was used for target protein determination, β-actin (81115-1-RR, dilution 1: 10000), and HRP-conjugated goat anti-rabbit IgG secondary antibody (SA00001-2, dilution 1: 10000) was used as loading control in this study. All antibodies were purchased from Proteintech Group, Inc. (Rosemont, USA).

### Gut microbiota analysis

The bacterial DNA from the cecal contents (8 samples/group) was extracted as previously described (20). One percent agarose gel was used to determine the quality of DNA samples. After measuring the concentration, ~20–30 ng of qualified DNA was used to prepare the library and subjected to Illumina sequencing performed by Meiji Biotechnology Co., Ltd (Shanghai, China). The V3 and V4 hypervariable microbial 16S rDNA regions were amplified by PCR using the following primers: 38F (5′-ACTCCTACGGGAGGCAGCAG-3′); 806R (5′-GGACTACHVGGGTWTCTAAT-3′). The PCR products were recycled using 2% agarose gel and purified using AxyPrep DNA Gel Extraction Kit (Axygen Biosciences, Union City, CA, USA). The library construction quality was checked using QuantiFluor™-ST (Promega, USA). The amplicon was sequenced using an Illumina MiSeq instrument (Illumina, San Diego, CA, USA), and the raw data were processed with QIIME (1.70) software. All raw data were filtered and trimmed by the Trimmomatic method. UPARSE software (version 7.1, http://drive5.com/uparse/) was used to cluster OTUs according to 97% similarity against the Silva_138 16SrRNA database (http://www.arbsilva.de/). ANOSIM and unweighted principal component analysis (PCoA) were used to compare the bacterial diversity between the control and treatment groups. The above 16S rRNA sequencing analysis was performed by Meiji Biotechnology Co., Ltd (Shanghai, China).

### Statistical analysis

All the statistical analyses were performed using GraphPad Prism 8.0 software (San Diego, CA, USA). All data were presented as mean ± SEM. The student's *t*-test was used to compare the treatments. A *p*-value ≤ 0.05 was considered significant. Correlations between the gut microbiota abundance and environmental factors were calculated using Pearson's correlation analysis. The PCoA analysis of bile acid was conducted in R using the permutational multivariate analysis of variance (PERMANOVA) program.

## Results

### Effect of chronic corticosterone exposure on the contents of TG and Chol in the liver

**Figure 1:**
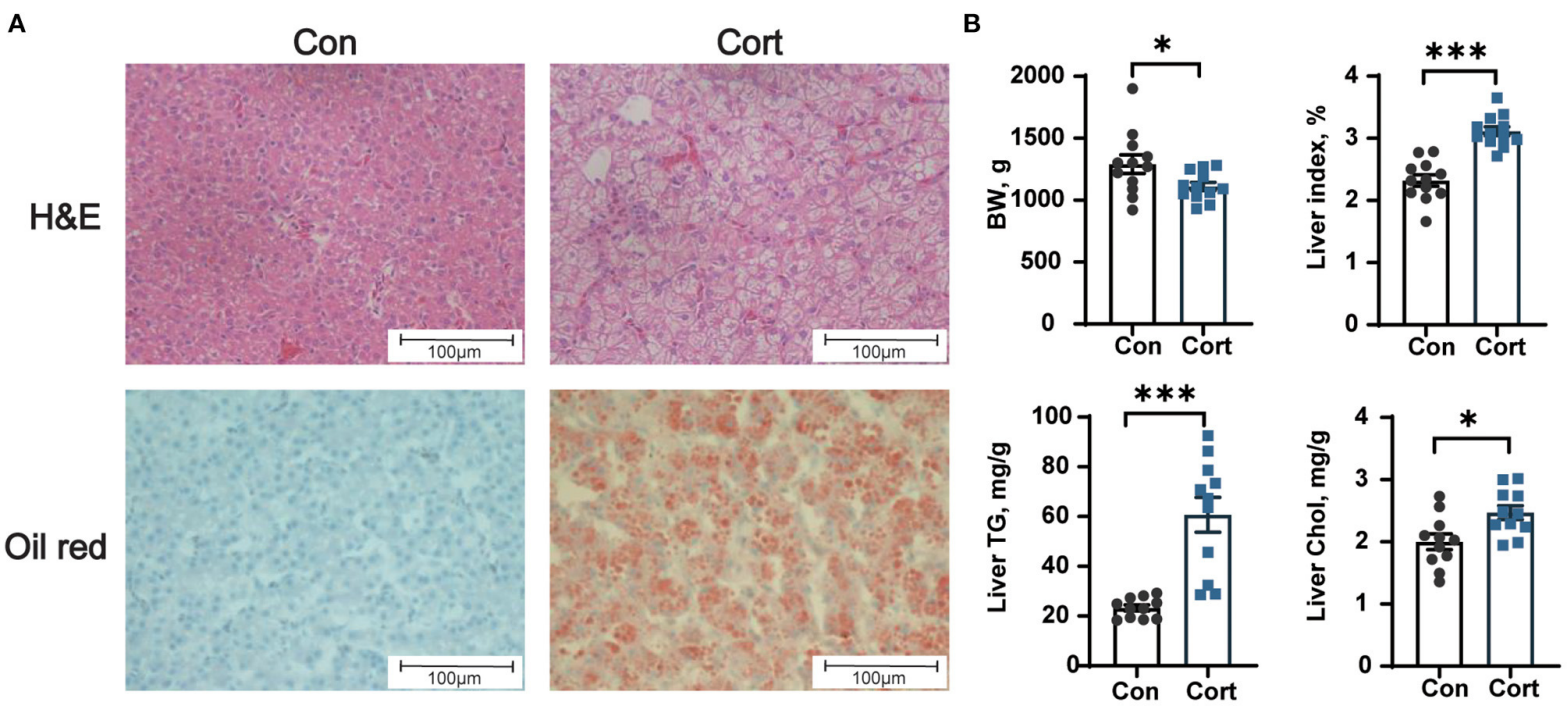
Effect of chronic corticosterone exposure on the contents of TG and Chol in the liver. **(A)** Hematoxylin and eosin and oil red staining; **(B)** Final body weight, liver index, TG, and Chol levels in the liver. Values are means ± SEM. *p < 0.05, ***p < 0.001 compared with control (*n* = 12) by student's *t*-test. Control = chicken injected with solvent (15% ethanol in saline); Cort = chicken injected with corticosterone (4.0 mg/kg body weight).

Chronic Cort exposure significantly elevated the concentrations of plasma TG (*p* < 0.05), Chol (*p* < 0.05), HDL (*p* < 0.05), LDL (*p* < 0.05), and Glu (*p* < 0.05, Table 1). Compared with the control group, there were obvious vacuoles in hepatocytes by HE staining and lots of lipid droplets were observed in hepatocytes by oil red staining (Figure 1A). Similar to the histological result, Cort exposure significantly increased the liver index (*p* < 0.05) and the contents of TG (*p* < 0.05) and Chol (*p* < 0.01) in the liver (Figure 1B).

**Table 1:** Effects of chronic corticosterone exposure on the profiles of plasma lipid and glucose levels in chickens.

**Serum parameters**	**Con**		**Cort**		***p*-value**
	**Mean**	**SEM**	**Mean**	**SEM**	
TG, mmol/L	0.76	0.35	4.34	0.56	0.032
Chol, mmol/L	2.84	0.74	5.43	1.02	0.001
HDL-C, mmol/L	2.37	0.56	3.07	0.69	0.033
LDL-C, mmol/L	0.87	0.18	1.53	0.25	0.001
Glu, mmol/L	12.70	2.67	29.82	2.68	0.022

### Effect of chronic corticosterone exposure on TBA levels in plasma, liver, and feces

**Figure 2:**
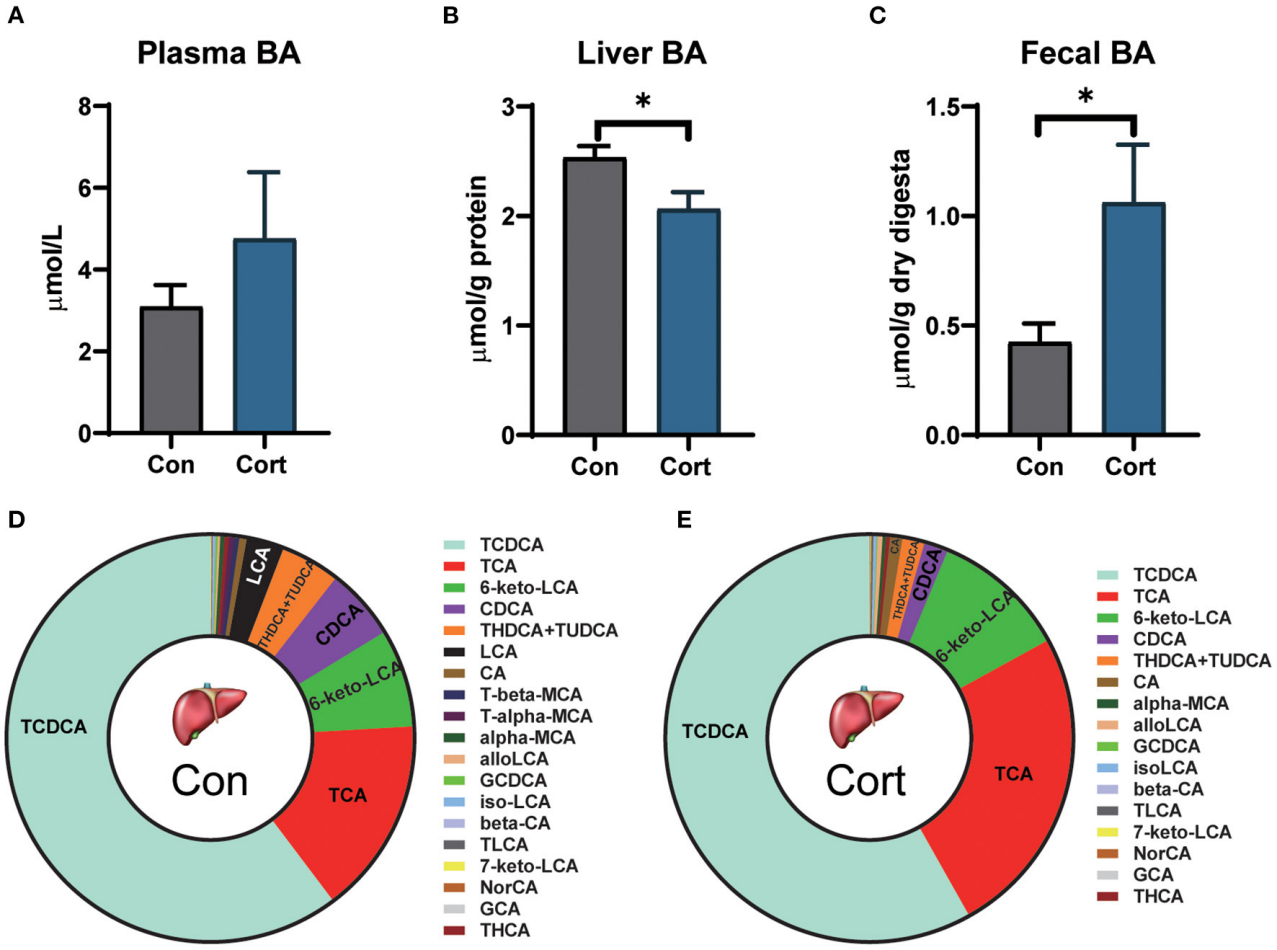
Effect of chronic corticosterone exposure on TBAs levels in plasma, liver, and feces. **(A)** Plasma TBAs; **(B)** Liver TBAs; **(C)** Fecal TBAs; Values are means ± SEM. *p < 0.05 compared with control (*n* = 12) by student's *t*-test. **(D, E)** Liver bile acid profiles in Con and Cort groups (*n* = 5). Control = chicken injected with solvent (15% ethanol in saline); Cort = chicken injected with corticosterone (4.0 mg/kg body weight).

Due to the importance of BA in lipid metabolism, the levels of TBA in plasma, liver, and feces were determined in Con and Cort chicken. It was shown that chronic Cort exposure didn't affect the plasma level of TBA (Figure 2A). However, Cort treatment significantly (*p* < 0.05) decreased the amount of TBA in the liver but increased the TBA in feces (Figures 2B, C).

To analyze the detailed changes, BA from the liver was extracted and profiled by UPLC-HRMS (Figures 2D, E). PCA analysis showed that the Cort group significantly separated from the Con group (*p* < 0.05; Supplementary Figure 1). Chronic Cort exposure significantly decreased the levels of CDCA, T-alpha-MCA, and T-beta-MCA (*p* < 0.01). The most remarkable change in content was CDCA, which decreased by 3.09 μg/g in the Cort group. In contrast, the TLCA was significantly increased in the Cort group (*p* < 0.05).

### Effect of chronic corticosterone exposure on the gene expression for BA synthesis and transportation in the liver and ileum

**Figure 3:**
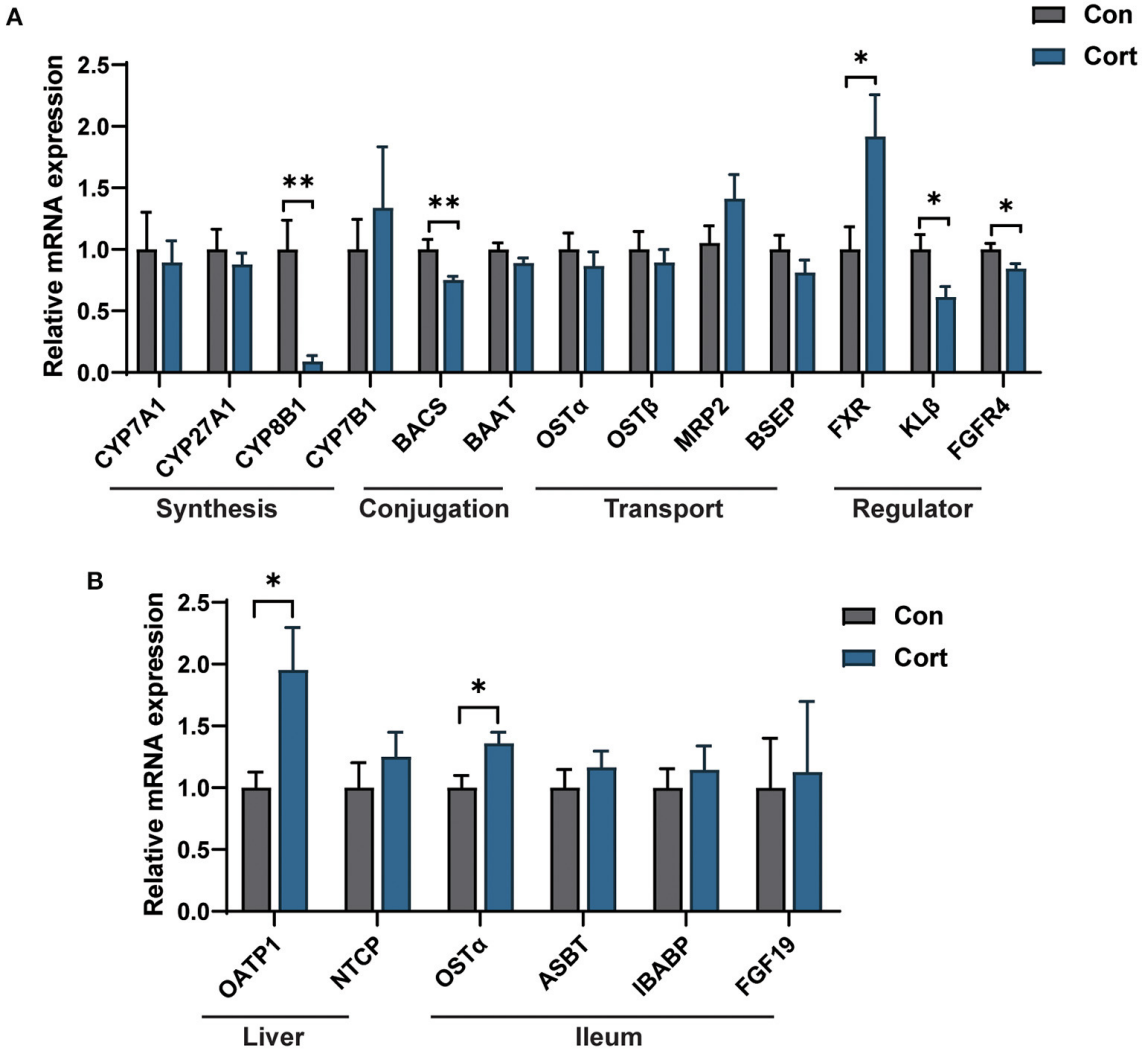
Effect of chronic corticosterone exposure on the gene expression for BA synthesis and transportation in the liver and ileum. **(A)** BA metabolism-related genes in the liver; **(B)** Selected genes related to BA enterohepatic circulation in the liver and ileum. Values are means ± SEM. *p < 0.05, **p < 0.01 compared with control (*n* = 12) by student's *t*-test.

It was observed that chronic Cort exposure significantly decreased the expression of CYP8B1 (*p* < 0.01), BACS (*p* < 0.01), KLβ (*p* < 0.05), and FGFR4 (*p* < 0.05), but significantly increased the expression of FXR (*p* < 0.05, Figure 3A) in the liver. Although no significant difference was found in CYP27A1 mRNA expression, its protein level was significantly decreased (*p* < 0.05, Supplementary Figure 2). In addition, it was found that chronic Cort exposure significantly increased the expression of OATP1 (*p* < 0.05), and OSTα (*p* < 0.05) in the ileum (Figure 3B).

### Chronic corticosterone exposure disrupts gut microbiota composition in chicken

**Figure 4:**
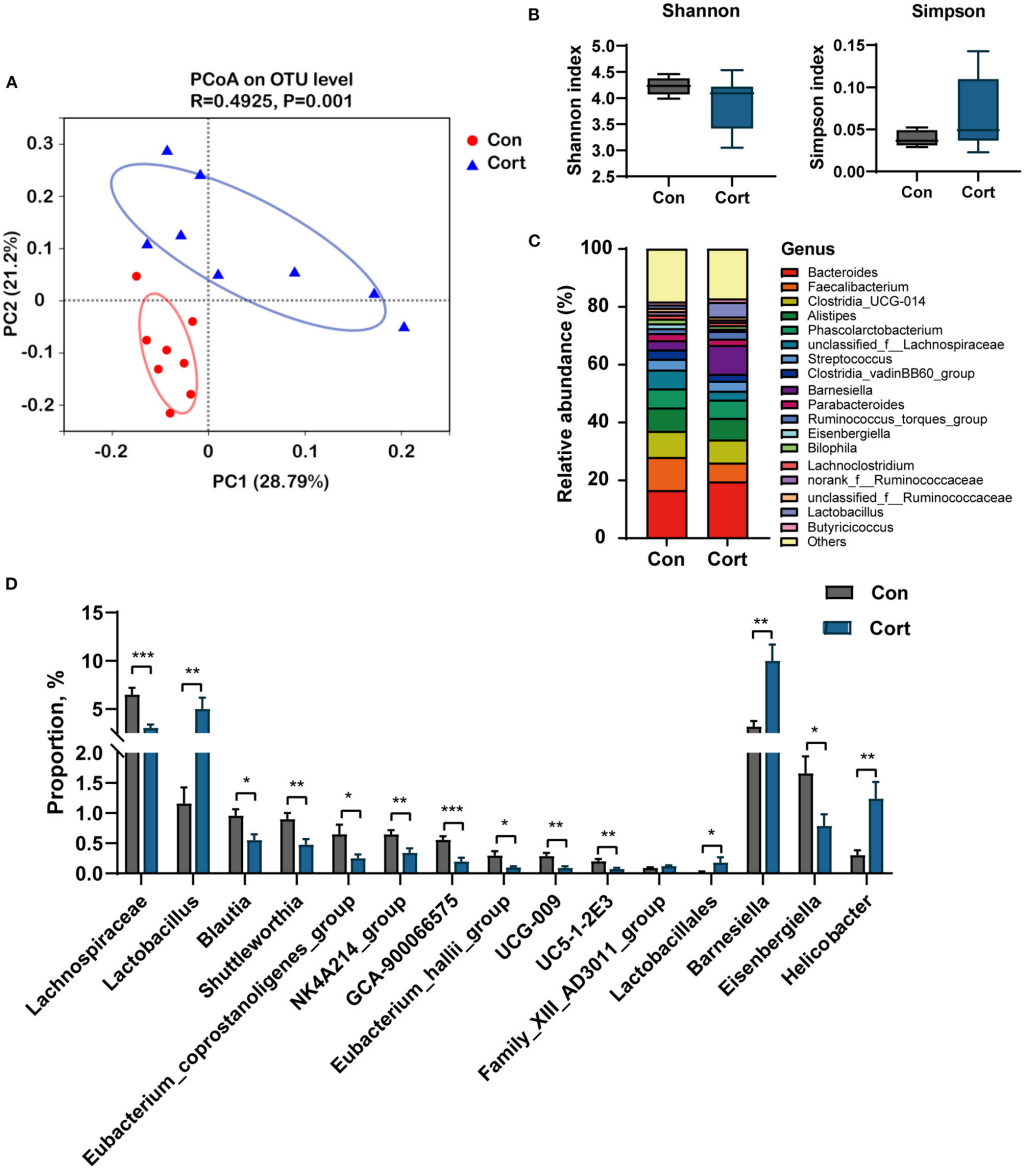
Chronic corticosterone exposure changes gut microbiota composition in chicken. **(A)** PCoA analysis between Con and Cort treatment; **(B)** Shannon and Simpson indexes; **(C)** Relative abundance of bacteria at genus level; **(D)** Relative abundance of bacteria associated with BA metabolism. Values are means ± SEM. *p < 0.05, **p < 0.01, ***p < 0.001 compared with control (*n* = 8) by student's *t*-test.

Bile acids are synthesized in the liver and metabolized in the gut by various microbes. As presented above, we have found an apparent change in the liver and fecal TBA upon Cort treatment. Thus, we investigated whether these changes might be related to alterations in gut microbiota composition. Results showed that Cort exposure significantly changed the gut microbiota composition compared to the Con group (Figure 4A). Cort treatment didn't affect the phylotype richness indexes, such as the Shannon and Simpson indexes (Figure 4B). At the genus level, we found that the abundance of *Barnesiella, Lactobacillus*, and *Helicobacter* was significantly increased in Cort. And we also found that *Lachnospiraceae, Eisenbergiella, Blautia*, and *Eubacterium* decreased considerably upon Cort treatment (Figure 4C). Among the 15 genera significantly modified under Cort, 10 were reduced, with the other five microbes increased (Figure 4D). Therefore, the above results indicated that Cort exposure altered the gut microbiota composition, which might impact the BA metabolism.

**Figure 5:**
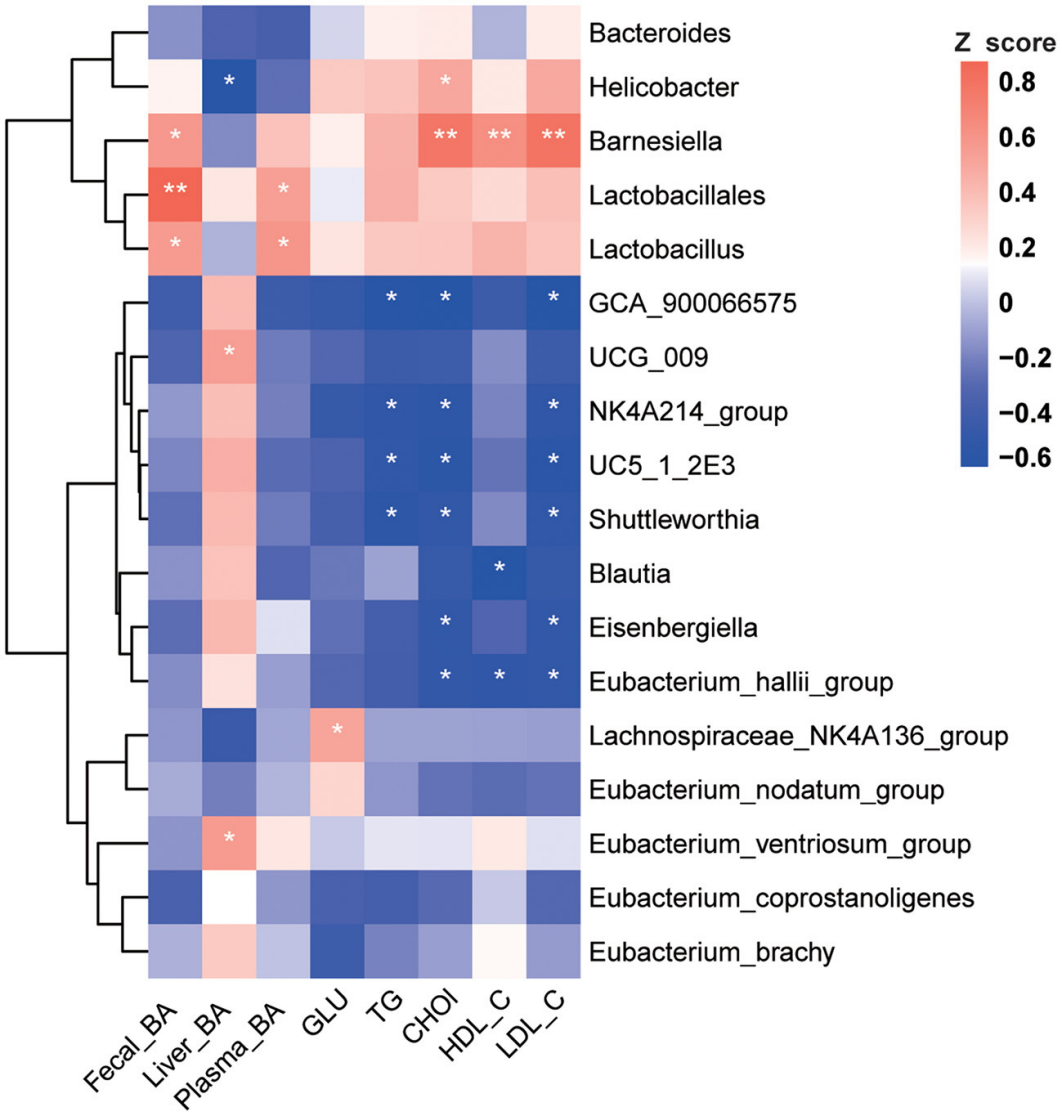
Correlation analysis between environmental factors and BA-associated bacteria. Pearson's correlation coefficients between the relative abundances of the significantly changed BA-related bacterial and clinical metabolic parameters (fecal BA, liver BA, plasma BA, GLU, TG, Chol, HDL, and LDL). *p < 0.05, **p < 0.01 indicate significant correlations. Blue color, negative correlation; Red color, positive correlation (*n* = 8).

Spearman correlation analysis was performed on significantly changed microbes and metabolic profiles (fecal TBA, liver TBA, plasma TBA, Glu, TG, Chol, HDL, and LDL). Results showed a significant positive correlation between fecal TBA and the abundance of *Lactobacillus* (*p* < 0.05), *Lactobacillales* (*p* < 0.01), and *Barnesiella* (*p* < 0.05). TBA in the liver was positively correlated with *Eubacterium* (*p* < 0.05) and negatively correlated with *Helicobacter* (*p* < 0.05). Plasma Chol was positively correlated with *Lactobacillus* and *Barnesiella* (*p* < 0.05) and negatively correlated with *Blautia* and *Eubacterium* (*p* < 0.05, Figure 5).

## Discussion

The intensive farm system causes chickens to be under various stress, leading to high-circulated GC. Previous studies showed that an excessive amount of GC induced fatty liver by promoting the expression of lipid synthesis genes in the chicken liver (2). In this current study, long-term treatment with GC successfully elevated the amount of lipids in both circulation and the liver. Due to the unique metabolic effect of bile acids on lipids, we conducted a comprehensive analysis of BA biosynthesis and enterohepatic circulation in this study. The results showed no significant change in serum BA after chronic stress; however, BA content in the liver decreased significantly, and BA in the cecum increased significantly. The targeted metabolomics analysis of BA in the liver showed that the content of primary bile acid CDCA decreased significantly. These results indicate that chronic GC exposure seriously destroys the metabolic homeostasis of BAs.

BAs are endogenous ligands that activate different nuclear and membrane receptors, such as FXR and TGR5, to regulate hepatic lipid, glucose, and energy metabolism (21). Compared with the previous results, we found that the hepatic expression of *FXR* was significantly up-regulated in the Cort group. In contrast, the expression of *CYP8B1* and *CYP27A1* was downregulated considerably in the liver under chronic stress. Chronic stress also promoted the expression of bile acid transporters, *NTCP* and *OATP1*, in enterohepatic circulation. Similar to previous studies, chronic heat stress can downregulate the expression of *CYP27A1* (22), an essential gene in the alternative bile acid synthesis pathway (23). Moreover, the expression of bile acid-binding transporters *NTCP, OATP1*, and *BSEP* was also inhibited in the Cort group. Research has shown that GR functions to promote the gene expression of NTCP in the presence of its ligand dexamethasone (24). According to the above studies, chronic GC exposure might increase the expression of NTCP and OATP1 to facilitate the reabsorption of BA from enterohepatic circulation, which further activates the FXR to inhibit the *de novo* synthesis of BA in the liver.

Besides being a hepatic metabolic regulator, BA is also an essential regulator of the gut microbiome. The unique chemical properties of BA can affect the composition and distribution of the gut microbiome. Meanwhile, the gut microbiome functions to hydrolyze the BA, which further affects the homeostasis of the bile acid pool and enterohepatic circulation (25, 26). The results of this study showed that after chronic corticosterone exposure, the enrichment of *Barnesiellaceae* and *Lactobacillaceae* increased significantly (*p* < 0.05), while B*lautia* and *Eubacterium* decreased significantly (*p* < 0.05). A recent review provides a broad insight into microbiome signatures for human NAFLD and explores issues with disentangling these signatures from underlying metabolic disorders (27). Additionally, Gao et al. have reported that chronic stress promotes colitis by disturbing the gut microbiota and triggering immune system response (28). Studies showed that *Lactobacillus, Clostridium, Escherichia*, and *Streptococcus* were enriched considerably in NAFLD patients. Differently, the abundance of *Blautia* decreased significantly in NASH patients (29, 30). Correlation analysis showed that under chronic corticosterone exposure, the enrichment of *Lactobacillaceae* was positively related to the increase of TG, LDL, and Chol in plasma and TBA in feces (*p* < 0.05). In this current study, chronic corticosterone exposure induced LDL and Chol increase was negatively associated with *Blautia*, which plays a vital role in glucose metabolism and obesity-related inflammation (31, 32). Therefore, the above results indicate that chronic corticosterone treatment-induced gut microbiome dysbiosis could lead to the development of fatty liver in chickens.

Meanwhile, *Lactobacillus, Clostridium, Bacteroides*, and *Eubacterium* can uncouple primary bile acids and transform them into secondary bile acids through modification (33). The current results present a significant positive correlation (*p* < 0.05) between total bile acids in the liver and gut microbiome, indicating that chronic stress may affect the metabolism of bile acids in the liver through changes in the gut microbiome. Thus, chronic Cort exposure may cause gut microbiome dysbiosis *via* modulation of bile acids. Moreover, the structural changes of intestinal flora, such as the enrichment of BSH microbiome species, may further alter the homeostasis of bile acid metabolism. In conclusion, the interaction between bile acids and the gut microbiome under chronic Cort exposure was related to the development of fatty liver in chickens.

## Conclusions

This study indicates that chronic Cort exposure induces fatty liver in chicken, which disrupts the recycling and synthesis of bile acids in the liver, resulting in a decrease in liver TBA. In addition, the elevated amount of fecal TBA changed the gut microbial composition. The significant change in *Lactobacillus* was related to fecal bile acids, TG, and Chol, and the change in *Eubacterium* was associated with liver bile acids. Further studies are needed to evaluate the function of specific BA and gut microbiome species in preventing chronic stress-induced metabolic disorders.

## Data availability statement

The data presented in the study are deposited in the NCBI sequence read archive (SRA) repository, accession number PRJNA956664: https://www.ncbi.nlm.nih.gov/bioproject/PRJNA956664/.

## Ethics statement

The animal study was reviewed and approved by Animal Ethics Committee of Nanjing Agricultural University.

## Author contributions

LW and XL directed this project and prepared the manuscript. AZ and HC conducted the animal studies. The funding was acquisited by LW and YJ. YJ contributed to the experimental design and finalized the manuscript. All authors have read and agreed to the published version of the manuscript.

## Glossary

ASBT, the apical ileal sodium-dependent bile acid cotransporter; BA, bile acids; BAAT, bile acid-coenzyme A:amino acid N-acyltransferase; BACS, bile acid: CoA synthetase; BSEP, bile salt export pump; CA, cholic acid; CDCA, chenodeoxycholic acid; Cort: corticosterone; CYP7A1, cholesterol 7-α hydroxylase; CYP27A1, sterol 27-α hydroxylase; CYP7B1, oxysterol 7-α hydroxylase; CYP8B1, microsomal sterol 12-α hydroxylase; DCA, deoxycholic acid; FXR, farnesoid x receptor; GC, glucocorticoid; GCA, glycol-cholic acid; GCBA, glyco-conjugated bile acids; GCDCA, glycol-chenodeoxycholic acid; GHCA, glycol-hyocholic acid; GUDCA, glycolursodeoxycholic acid; HCA, hyocholic acid; HDCA, hyodeoxycholic acid; LCA, lithocholic acid; PCoA, principal coordinate analysis; TBA, total bile acids; TCA, tauro-cholic acid; TCBA, tauro-conjugated bile acids; TCDCA, tauro-chenodeoxycholic acid; TDCA, tauro-deoxycholic acid; TG, triglycerides; TGR5, Takeda G protein-coupled receptor 5; THDCA, tauro-hyodeoxycholic acid; TLCA, tauro-lithocholic acid; TUDCA, tauro-ursodeoxycholic acid; UDCA, ursodeoxycholic acid; MRP2, multidrug resistance associated protein 2; OSTα/β, organic solute transporter α/β (OSTα/β).
