# Supplementary material for: Chronic corticosterone exposure disrupts hepatic and intestinal bile acid metabolism in chicken
Source: Front Vet Sci. 2023 May 17;10:1147024. doi: 10.3389/fvets.2023.1147024 (PMC10229839; doi:10.3389/fvets.2023.1147024)
Supplement: Supplementary file 1 [file Data_Sheet_1.docx]

Supplementary Material

Chronic corticosterone exposure disrupts hepatic and intestinal bile acid metabolism in chicken

Lei Wu^1^, Xinyi Liu^1^, Aijia Zhang^1^, Huimin Chen^1^, Ruqian Zhao^1^, Yimin Jia^1,2*^

^1^Key Laboratory of Animal Physiology & Biochemistry, College of Veterinary Medicine, Nanjing Agricultural University, Nanjing 210095, P. R. China.

^2^Jiangsu Collaborative Innovation Center of Meat Production and Processing, Quality and Safety Control, Nanjing 210095, P. R. China.

*** Correspondence:**Yimin Jia
jymrobin@hotmail.com

**Supplementary Figure 1.**


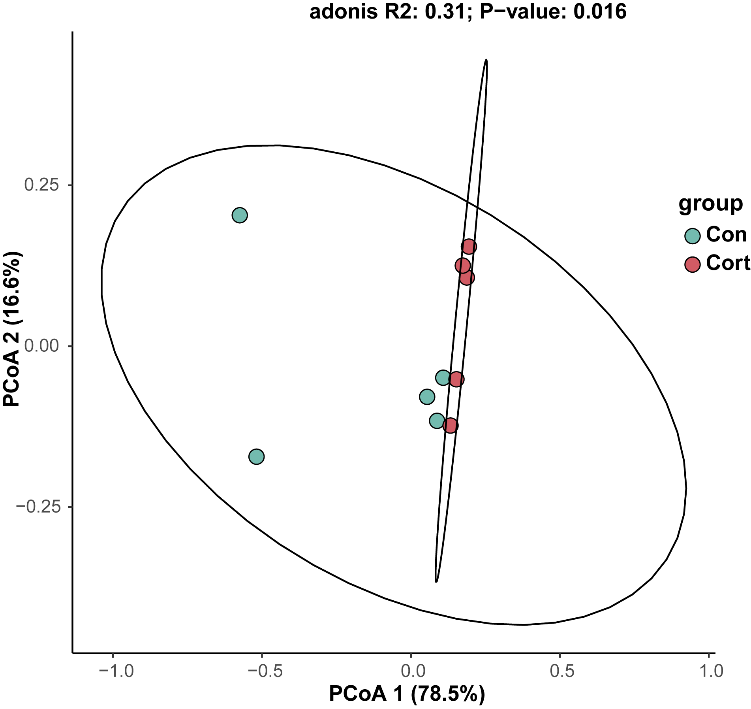


**Supplementary Figure 1. PCoA analysis of bile acid profiles between Con and Cort treatment.** PCoA: a principal component analysis. The PCoA analysis was conducted in R using the permutational multivariate analysis of variance (PERMANOVA) program.

**Supplementary Figure 2.**


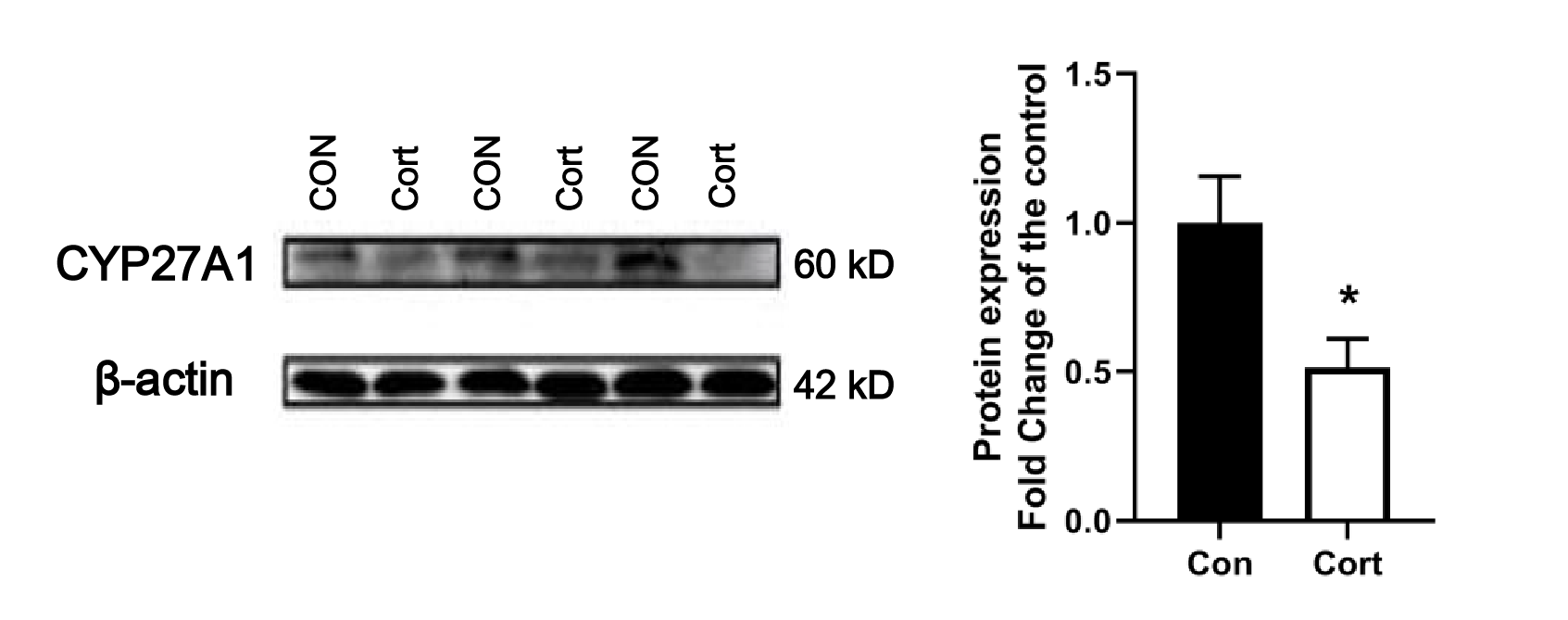


**Supplementary Figure 2. Cort exposure inhibits the protein expression of CYP27A1 in the liver.** Protein expression values are fold changes relative to the mRNA levels in the Con group. CYP27A1: sterol 27-hydroxylase. Values are means ± SEM, *P < 0.05 compared with control (n = 8) by student’s *t*-test.

**Supplementary Table 1.** **Primer sequence for RT-PCR**

| **Gene** | **Primer sequence** |
| --- | --- |
| **CYP7A1** | F: GTAACGCCCTAGATGCCCTC  R: GCTCTCTCTGTTTCCCGCTT |
| **CYP27A1** | F:TATCCCCAAGATGCCGATGC  R: TGGGGAAGAGGTAGTCTCCG |
| **CYP8B1** | F: GGGTTACGCACTGGACTTCA  R: GTAGCCAAAAACCCGGAGGA |
| **CYP7B1** | F: CAAAATGGCTGGGAGGGTCA  R: ATGGTCAATCTCGTCACGCA |
| **BAAT** | F:TGGACAACTCGGCCATCTTC  R:CACGAAGAGGACCTTGCCTT |
| **BACS** | F:CAGCTAGCTACTGCAGATGGA  R:CATCTGCCTCTTGCACTGGA |
| **OSTɑ** | F: GGCAGATGATCCCAGGTTCC  R: AGCTGGTCTTGCGGTAGATG |
| **OSTβ** | F: CTGCTGCAAGACACTGAAGC  R: CACAGGGACTTGTCTGCACT |
| **MRP2** | F: TCTGCTTGTGCAGAGACTCG  R: TACATCCACGATGGGGTCCT |
| **BSEP** | F: TGTTGGTGCTAGTGGAGCTG  R: TGCTGTGGCAAGTCCATGAT |
| **FXR** | F: TGGTCTGTGGTGACAAAGCC  R: ACATGCCCATTTGCTTGCAT |
| **KLβ** | F:CCACTGGATCGAACCCAACA  R:CTCTGGATAATCGCCGTCCC |
| **FGFR4** | F:GCACCGTACTGGACTCACC  R:GCAGGTAGCTGTAGCGGATG |
| **OATP1** | F: CGGAAACACCCAGCACAATG  R: AAGATCCAAGCAGGAAGCCC |
| **NTCP** | F: CCAATCCCTTCCTCACCTCG  R: GAGTGCAAATGGTGGTGCTG |
| **ASBT** | F: GGGGATGATGCCACTCTGTC  R: CCCCCAACCACAGCAGTAAT |
| **IBABP** | F:AGAAGATCGGTCTCCCTGCT  R:ATTAGTCGTGGTGCGTCCTC |
| **FGF19** | F: CCGCAGAGTCTGTTGGAGAT  R: GTTGTAGCCGTCTGGACGAA |
| **18S rRNA** | F: ATAACGAACGAGACTCTGGCA  R: CGGACATCTAAGGGCATCACA |
